# MDMA-assisted therapy for severe PTSD: a randomized, double-blind, placebo-controlled phase 3 study

**DOI:** 10.1038/s41591-021-01336-3

**Published:** 2021-05-10

**Authors:** Jennifer M. Mitchell, Michael Bogenschutz, Alia Lilienstein, Charlotte Harrison, Sarah Kleiman, Kelly Parker-Guilbert, Marcela Ot’alora G., Wael Garas, Casey Paleos, Ingmar Gorman, Christopher Nicholas, Michael Mithoefer, Shannon Carlin, Bruce Poulter, Ann Mithoefer, Sylvestre Quevedo, Gregory Wells, Sukhpreet S. Klaire, Bessel van der Kolk, Keren Tzarfaty, Revital Amiaz, Ray Worthy, Scott Shannon, Joshua D. Woolley, Cole Marta, Yevgeniy Gelfand, Emma Hapke, Simon Amar, Yair Wallach, Randall Brown, Scott Hamilton, Julie B. Wang, Allison Coker, Rebecca Matthews, Alberdina de Boer, Berra Yazar-Klosinski, Amy Emerson, Rick Doblin

**Affiliations:** 1 Department of Neurology, University of California San Francisco, San Francisco, CA USA; 2 Department of Psychiatry and Behavioral Sciences, University of California San Francisco, San Francisco, CA USA; 3 Department of Psychiatry, New York University Grossman School of Medicine, New York, NY USA; 4 Multidisciplinary Association for Psychedelic Studies (MAPS), San Jose, CA USA; 5 MAPS Public Benefit Corporation (MAPS PBC), San Jose, CA USA; 6 Kleiman Consulting and Psychological Services, Sayreville, NJ USA; 7 KPG Psychological Services LLC, Brunswick, ME USA; 8 Aguazul-Bluewater Inc., Boulder, CO USA; 9 MDMA Therapy Training Program, MAPS Public Benefit Corporation, San Jose, CA USA; 10 Nautilus Sanctuary, New York, NY USA; 11 Fluence, Woodstock, NY USA; 12 Department of Family Medicine and Community Health, University of Wisconsin School of Medicine and Public Health, Madison, WI USA; 13 Medical University of South Carolina, Charleston, SC USA; 14 San Francisco Insight and Integration Center, San Francisco, CA USA; 15 British Columbia Centre on Substance Use, Vancouver, British Columbia Canada; 16 Boston University School of Medicine, Boston, MA USA; 17 Chaim Sheba Medical Center, Tel HaShomer, Israel; 18 Ray Worthy Psychiatry LLC, New Orleans, LA USA; 19 Wholeness Center, Fort Collins, CO USA; 20 New School Research LLC, North Hollywood, CA USA; 21 Zen Therapeutic Solutions, Mt Pleasant, SC USA; 22 University of Toronto, Toronto, Ontario Canada; 23 Dr Simon Amar Inc., Montreal, Quebec Canada; 24 Be’er Ya’akov Ness Ziona Mental Health Center, Be’er Ya’akov, Israel; 25 Stanford School of Medicine, Stanford, CA USA

**Keywords:** Drug development, Phase III trials

## Abstract

Post-traumatic stress disorder (PTSD) presents a major public health problem for which currently available treatments are modestly effective. We report the findings of a randomized, double-blind, placebo-controlled, multi-site phase 3 clinical trial (NCT03537014) to test the efficacy and safety of 3,4-methylenedioxymethamphetamine (MDMA)-assisted therapy for the treatment of patients with severe PTSD, including those with common comorbidities such as dissociation, depression, a history of alcohol and substance use disorders, and childhood trauma. After psychiatric medication washout, participants (*n* = 90) were randomized 1:1 to receive manualized therapy with MDMA or with placebo, combined with three preparatory and nine integrative therapy sessions. PTSD symptoms, measured with the Clinician-Administered PTSD Scale for DSM-5 (CAPS-5, the primary endpoint), and functional impairment, measured with the Sheehan Disability Scale (SDS, the secondary endpoint) were assessed at baseline and at 2 months after the last experimental session. Adverse events and suicidality were tracked throughout the study. MDMA was found to induce significant and robust attenuation in CAPS-5 score compared with placebo (*P* < 0.0001, *d* = 0.91) and to significantly decrease the SDS total score (*P* = 0.0116, *d* = 0.43). The mean change in CAPS-5 scores in participants completing treatment was −24.4 (s.d. 11.6) in the MDMA group and −13.9 (s.d. 11.5) in the placebo group. MDMA did not induce adverse events of abuse potential, suicidality or QT prolongation. These data indicate that, compared with manualized therapy with inactive placebo, MDMA-assisted therapy is highly efficacious in individuals with severe PTSD, and treatment is safe and well-tolerated, even in those with comorbidities. We conclude that MDMA-assisted therapy represents a potential breakthrough treatment that merits expedited clinical evaluation.

## Abstract (web summary)

Results from a phase 3, double-blind, randomized, placebo-controlled trial demonstrate that MDMA-assisted therapy is safe and effective in treating severe post-traumatic stress disorder.

## Main

PTSD is a common and debilitating condition with immeasurable social and economic costs that affects the lives of hundreds of millions of people annually. There are a number of environmental and biological risk factors that contribute to the development and maintenance of PTSD^1^, and poor PTSD treatment outcomes are associated with several comorbid conditions that include childhood trauma^2^, alcohol and substance use disorders^3^, depression^4^, suicidal ideation^5^ and dissociation^6^. It is therefore imperative to identify a therapeutic that is beneficial in those individuals with the comorbidities that typically confer treatment resistance.

The selective serotonin reuptake inhibitors (SSRIs) sertraline and paroxetine are Food and Drug Administration (FDA)-approved first-line therapeutics for the treatment of PTSD. However, an estimated 40–60% of patients do not respond to these compounds^7^. Likewise, although evidenced-based trauma-focused psychotherapies such as prolonged exposure and cognitive behavioral therapy are considered to be the gold standard treatments for PTSD^8^, many participants fail to respond or continue to have significant symptoms, and dropout rates are high^9,10^. Novel cost-effective therapeutics are therefore desperately needed^11^.

The substituted amphetamine 3,4-methylenedioxymethamphetamine (MDMA) induces serotonin release by binding primarily to presynaptic serotonin transporters^12^. MDMA has been shown to enhance fear memory extinction, modulate fear memory reconsolidation (possibly through an oxytocin-dependent mechanism), and bolster social behavior in animal models^13,14^. Pooled analysis of six phase 2 trials of MDMA-assisted therapy for PTSD have now shown promising safety and efficacy findings^15^.

Here, we assess the efficacy and safety of MDMA-assisted therapy in individuals with severe PTSD. Participants were given three doses of MDMA or placebo in a controlled clinical environment and in the presence of a trained therapy team. Primary and secondary outcome measures (CAPS-5 and SDS, respectively) were assessed by a centralized pool of blinded, independent diagnostic assessors. MDMA-assisted therapy for PTSD was granted an FDA Breakthrough Therapy designation, and the protocol and statistical analysis plan (SAP) were developed in conjunction with the FDA^16^.

## Results

### Demographics

**Fig. 1:**
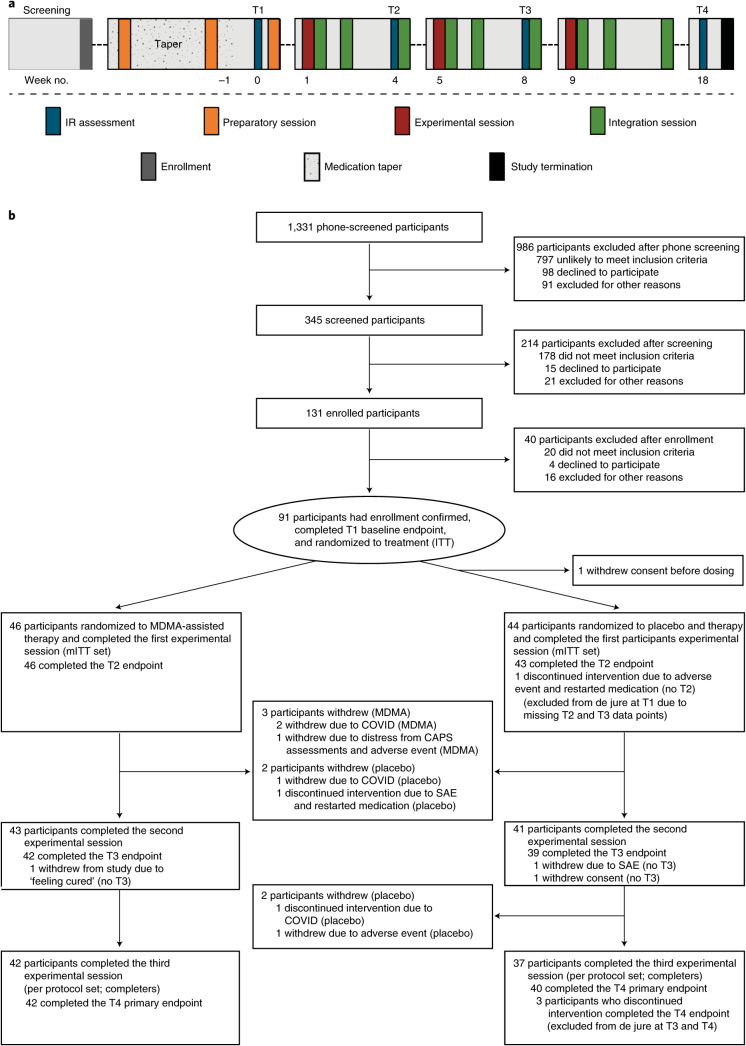
Procedure timeline and study flow diagram. **a**, Procedure timeline. Following the screening procedures and medication taper, participants attended a total of three preparatory sessions, three experimental sessions, nine integration sessions and four endpoint assessments (T1–4) over 18 weeks, concluding with a final study-termination visit. IR, independent rater; T, timepoint of endpoint assessment; T1, baseline; T2, after the first experimental session; T3, after the second experimental session; T4, 18 weeks after baseline. **b**, CONSORT diagram indicating participant numbers and disposition through the course of the trial.

Participants were recruited from 7 November 2018 to 26 May 2020, with the last participant visit conducted on 21 August 2020. A total of 345 participants were assessed for eligibility, 131 were enrolled, 91 were confirmed for randomization (United States, *n* = 77; Canada, *n* = 9; Israel, *n* = 5), and 46 were randomized to MDMA and 44 to placebo (Fig. 1).

Study arms were not significantly different in terms of race, ethnicity, sex, age, dissociative subtype, disability or CAPS-5 score (Table 1). The mean duration of PTSD diagnosis was 14.8 (s.d. 11.6) years and 13.2 (s.d. 11.4) years in the MDMA and placebo groups, respectively. Of note, six participants in the MDMA group and 13 participants in the placebo group had the dissociative subtype according to CAPS-5 score.

**Table 1:** Demographics and baseline characteristics

	MDMA-assisted therapy (*n* = 46)	Placebo with therapy (*n* = 44)	Total (*n* = 90)
Age (years), mean (s.d.)	43.5 (12.9)	38.2 (10.4)	41.0 (11.9)
Sex assigned at birth, *n* (%)			
Male	19 (41.3)	12 (27.3)	31 (34.4)
Female^a^	27 (58.7)	32 (72.7)	59 (65.6)
Ethnicity, *n* (%)			
Hispanic or Latino	5 (10.9)	3 (6.8)	8 (8.9)
Not Hispanic or Latino	41 (89.1)	40 (90.9)	81 (90.0)
Race, *n* (%)			
American Indian or native Alaskan	3 (6.5)	0 (0.0)	3 (3.3)
Asian	2 (4.3)	5 (11.4)	7 (7.8)
Black or African American	0 (0.0)	2 (4.5)	2 (2.2)
Native Hawaiian or other Pacific Islander	0 (0.0)	0 (0.0)	0 (0.0)
White	39 (84.8)	30 (68.2)	69 (76.7)
Multiple	2 (4.3)	6 (13.6)	8 (8.9)
BMI (kg m^−2^), mean (s.d.)	26.0 (4.8)	24.8 (4.2)	25.4 (4.5)
Duration of PTSD (years), mean (s.d.)	14.8 (11.6)	13.2 (11.4)	14.1 (11.5)
Dissociative subtype of PTSD, *n* (%)	6 (13.0)	13 (29.5)	19 (21.1)
Comorbid major depression, *n* (%)	42 (91.3)	40 (90.9)	82 (91.1)
Veteran	10 (21.7)	6 (13.6)	16 (17.8)
Trauma history, *n* (%)			
Developmental trauma	40 (87.0)	36 (81.8)	76 (84.4)
Combat exposure	6 (13.0)	5 (11.4)	11 (12.2)
Multiple trauma	41 (89.1)	38 (86.4)	79 (87.8)
Pre-study PTSD medications, *n* (%)^b^			
Sertraline	8 (17.4)	9 (20.5)	17 (18.9)
Paroxetine	3 (6.5)	3 (6.8)	6 (6.7)
Pre-study therapy, *n* (%)			
CBT	12 (26.1)	22 (50.0)	34 (37.8)
EMDR	17 (37.0)	13 (29.5)	30 (33.3)
Group therapy	19 (41.3)	14 (31.8)	33 (36.7)
Prolonged exposure therapy	1 (2.2)	0 (0)	1 (1.1)
Psychodynamic	11 (23.9)	10 (22.7)	21(23.3)
Other	41 (89.1)	38 (86.4)	79 (87.8)
None	1 (2.2)	1 (2.3)	2 (2.2)
Baseline CAPS-5 total score, mean (s.d.)	44.0 (6.01)	44.2 (6.15)	44.1 (6.04)
Baseline SDS modified score, mean (s.d.)	6.8 (2.07)	7.4 (1.63)	7.1 (1.9)
Lifetime C-SSRS, *n* (%)^c^			
Positive lifetime suicidal ideation	42 (91.3)	41 (93.2)	83 (92.2)
Serious lifetime suicidal ideation	20 (43.5)	17 (38.6)	37 (41.1)
Positive lifetime suicidal behavior	16 (34.8)	13 (29.5)	29 (32.2)
Baseline BDI-II total score, mean (s.d.)	30.5 (13.1)	34.9 (12.6)	32.7 (13.0)
AUDIT, mean (s.d.)	4.1 (4.2)	2.8 (3.2)	3.5 (3.8)
DUDIT, mean (s.d.)	2.7 (4.3)	3.5 (4.5)	3.1 (4.4)
ACE Questionaire score, mean (s.d.)	5.0 (2.7)	5.0 (2.9)	5.0 (2.8)
Prior report of MDMA use, *n* (%)			
Lifetime reported use	18 (39.1)	11 (25.0)	29 (32.2)
Reported use in the past 10 years	9 (19.6)	10 (22.7)	19 (21.1)

BMI, body mass index; CBT, cognitive behavioral therapy; EMDR, eye movement desensitization and reprocessing therapy.

^a^Two participants included in the assigned female at birth MDMA group identified their gender as non-binary.

^b^Medications were tapered down and washed out prior to baseline assessments and the first experimental session, in accordance with the protocol.

^c^Lifetime accounts for all suicidal ideation and behavior prior to the study. Serious ideation is defined as a score of 4 or 5 in the suicidal ideation category.

### Efficacy

**Fig. 2:**
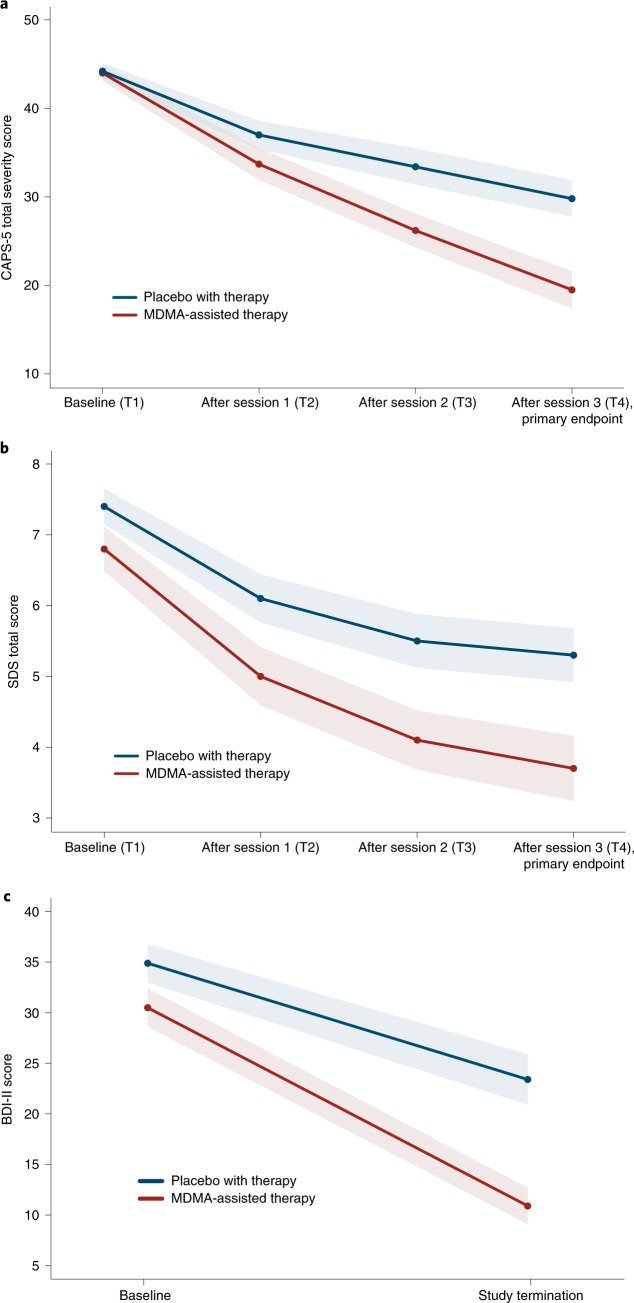
Measures of MDMA efficacy in the MDMA-assisted therapy group and the placebo group. **a**, Change in CAPS-5 total severity score from T1 to T4 (*P* < 0.0001, *d* = 0.91, *n* = 89 (MDMA *n* = 46)), as a measure of the primary outcome. Primary analysis was completed using least square means from an MMRM model. **b**, Change in SDS total score from T1 to T4 (*P* = 0.0116, *d* = 0.43, *n* = 89 (MDMA *n* = 46)), as a measure of the secondary outcome. Primary analysis was completed using least square means from an MMRM model. **c**, Change in BDI-II score from T1 to study termination (*t* = −3.11, *P* = 0.0026, *n* = 81 (MDMA *n* = 42)), as a measure of the exploratory outcome. Data are presented as mean and s.e.m.

MDMA significantly attenuated PTSD symptomology, as shown by the change in CAPS-5 total severity score from baseline to 18 weeks after baseline. Mixed model repeated measure (MMRM) analysis of the de jure estimand (that is, the effects of the drug if taken as directed) showed a significant difference in treatment arms (*n* = 89 (MDMA *n* = 46), *P* < 0.0001, between-group difference = 11.9, 95% confidence interval (CI) = 6.3–17.4, d.f. = 71) (Fig. 2a). MMRM sensitivity analysis of the de facto estimand (that is, the effects of the drug if taken as assigned, regardless of adherence) showed a significant difference in treatment arms (*n* = 90, *P* < 0.0001, d.f. = 72).

The mean change in CAPS-5 scores from baseline to 18 weeks after baseline in the completers (per protocol set) was −24.4 (s.d. 11.6) (*n* = 42) in the MDMA-assisted therapy group compared with −13.9 (s.d. 11.5) (*n* = 37) in the placebo with therapy group.

The effect size of the MDMA-assisted therapy treatment compared with placebo with therapy was *d* = 0.91 (95% CI = 0.44–1.37, pooled s.d. = 11.55) in the de jure estimand and *d* = 0.97 (95% CI = 0.51–1.42) in the de facto estimand. When the within-group treatment effect (which included the effect of the supportive therapy that was administered in both arms) was compared between the MDMA and placebo groups, the effect size was 2.1 (95% CI = −5.6 to 1.4) in the MDMA group and 1.2 (95% CI = −4.9 to 2.5) in the placebo group.

Over the same period, MDMA significantly reduced clinician-rated functional impairment as assessed with the SDS. MMRM analysis of the de jure estimand showed a significant difference in treatment arms (*n* = 89 (MDMA *n* = 46), *P* = 0.0116, d.f. = 71, effect size = 0.43, 95% CI = −0.01 to 0.88, pooled s.d. = 2.53) (Fig. 2b). The mean change in SDS scores from baseline to 18 weeks after baseline in the completers was −3.1 (s.d. 2.6) (*n* = 42) in the MDMA-assisted therapy group and −2.0 (s.d. 2.4) (*n* = 37) in the placebo with therapy group.

MDMA was equally effective in participants with comorbidities that are often associated with treatment resistance. Participants with the dissociative subtype of PTSD who received MDMA-assisted therapy had significant symptom reduction on the CAPS-5 (mean MDMA Δ = −30.8 (s.d. 9.0), mean placebo Δ = −12.8 (s.d. 12.8)), and this was similar to that in their counterparts with non-dissociative PTSD (mean MDMA Δ = −23.6 (s.d. 11.7), mean placebo Δ = −14.3 (s.d. 11.2)). The benefit of MDMA therapy was not modulated by history of alcohol use disorder, history of substance use disorder, overnight stay or severe childhood trauma. Results were consistent across all 15 study sites with no effect by study site (*P* = 0.1003). In MMRM analysis there was no obvious impact of SSRI history on effectiveness of MDMA (Supplementary Table 2).

MDMA therapy was effective in an exploratory endpoint analysis of the reduction of depression symptoms (using the Beck Depression Inventory II (BDI-II)) from baseline to study termination of the de jure estimand (mean MDMA Δ = −19.7 (s.d. 14.0), *n* = 42; mean placebo Δ = −10.8 (s.d. 11.3), *n* = 39; *t* = −3.11, *P* = 0.0026, d.f. = 79, effect size = 0.67, 95% CI = 0.22–1.12) (Fig. 2c).

**Fig. 3:**
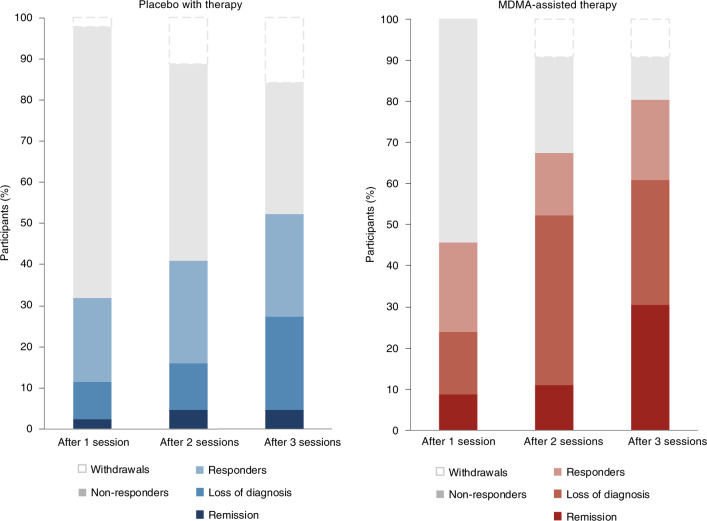
Treatment response and remission for MDMA and placebo groups as a percentage of total participants randomized to each arm (MDMA, *n* = 46; placebo, *n* = 44). Responders (clinically significant improvement, defined as a ≥10-point decrease on CAPS-5), loss of diagnosis (specific diagnostic measure on CAPS-5), and remission (loss of diagnosis and a total CAPS-5 score of ≤11) were tracked in both groups. Non-response is defined as a <10-point decrease on CAPS-5. Withdrawal is defined as a post-randomization early termination.

Clinically significant improvement (a decrease of ≥10 points on the CAPS-5), loss of diagnosis (specific diagnostic measure on the CAPS-5), and remission (loss of diagnosis and a total CAPS-5 score ≤ 11) were each tracked. At the primary study endpoint (18 weeks after baseline), 28 of 42 (67%) of the participants in the MDMA group no longer met the diagnostic criteria for PTSD, compared with 12 of 37 (32%) of those in the placebo group after three sessions. Additionally, 14 of 42 participants in the MDMA group (33%) and 2 of 37 participants in the placebo group (5%) met the criteria for remission after three sessions (Fig. 3).

### Safety

Treatment-emergent adverse events (TEAEs, adverse events that occurred during the treatment period from the first experimental session to the last integration session) that were more prevalent in the MDMA study arm were typically transient, mild to moderate in severity, and included muscle tightness, decreased appetite, nausea, hyperhidrosis and feeling cold (Supplementary Table 3). Importantly, no increase in adverse events related to suicidality was observed in the MDMA group. A transient increase in vital signs (systolic and diastolic blood pressure and heart rate) was observed in the MDMA group (Supplementary Table 4). Two participants in the MDMA group had a transient increase in body temperature to 38.1 °C: one had an increase after the second MDMA session, and one had an increase after the second and third MDMA sessions.

Two participants, both randomized to the placebo group, reported three serious adverse events (SAEs) during the trial. One participant in the placebo group reported two SAEs of suicidal behavior during the trial, and another participant in the placebo group reported one SAE of suicidal ideation that led to self-hospitalization. Five participants in the placebo group and three participants in the MDMA group reported adverse events of special interest (AESIs) of suicidal ideation, suicidal behavior or self-harm in the context of suicidal ideation. One participant in the placebo group reported two cardiovascular AESIs in which underlying cardiac etiology could not be ruled out (Table 2). One participant randomized to the MDMA group chose to discontinue participation due to being triggered by the CAPS-5 assessments and to an adverse event of depressed mood following an experimental session; this participant met the criterion as a non-responder, which was defined as having a less than 10-point decrease in CAPS-5 score. MDMA sessions were not otherwise followed by a lowering of mood.

**Table 2:** Participants with treatment-emergent SAEs and AESIs

	MDMA (*n* = 46), *n* (%)	Placebo (*n* = 44), *n* (%)
SAEs	–	2 (4.5)
Suicide attempts	–	1 (2.3)
Suicidal ideation resulting in self-hospitalization	–	1 (2.3)
AESIs		
Suicidality (total)	3 (6.5)	5 (11.4)
Suicidal ideation	2 (4.3)	3 (6.8)
Intentional self-harm in the context of suicidal ideation	1 (2.2)	–
Suicidal behavior (suicide attempts and preparatory acts) and self-harm	–	1 (2.3)
Suicidal behavior (preparatory acts), self-harm and suicidal ideation	–	1 (2.3)
Cardiac events that could indicate QT prolongation (total)	–	1 (2.3)
Irregular heartbeats and palpitations	–	1 (2.3)
Abuse potential for MDMA (total)	–	–

The number of participants experiencing one or more SAEs or AESIs relating to suicidality, cardiovascular symptoms that could indicate QT prolongation, and abuse potential following the first experimental session.

**Fig. 4:**
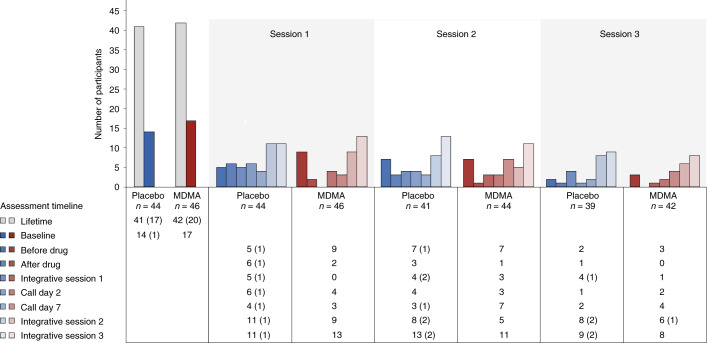
Number of participants reporting the presence of suicidal ideation as measured with the C-SSRS at each visit and separated by treatment group. C-SSRS ideation scores range from 0 (no ideation) to 5. A C-SSRS ideation score of 4 or 5 is termed ‘serious ideation’. The number of participants endorsing any positive ideation (>0) is shown by the colored bars and noted in the table below the graph. The number of participants endorsing serious ideation is given in parentheses in the table.

Suicidality was tracked throughout the study using the Columbia Suicide Severity Rating Scale (C-SSRS) at each study visit. More than 90% of participants reported suicidal ideation in their lifetime, and 17 of 46 participants (37%) in the MDMA group and 14 of 44 participants (32%) in the placebo group reported suicidal ideation at baseline. Although the number of participants who reported suicidal ideation varied throughout the visits, prevalence never exceeded baseline and was not exacerbated in the MDMA group. Serious suicidal ideation (a score of 4 or 5 on the C-SSRS) was minimal during the study and occurred almost entirely in the placebo arm (Fig. 4).

## Discussion

Here, we demonstrate that three doses of MDMA given in conjunction with manualized therapy over the course of 18 weeks results in a significant and robust attenuation of PTSD symptoms and functional impairment as assessed using the CAPS-5 and SDS, respectively. MDMA also significantly mitigated depressive symptoms as assessed using the BDI-II. Of note, MDMA did not increase the occurrence of suicidality during the study.

These data illustrate the potential benefit of MDMA-assisted therapy for PTSD over the FDA-approved first-line pharmacotherapies sertraline and paroxetine, which have both exhibited smaller effect sizes in pivotal studies^16^. Previous comparison of change in CAPS score between sertraline and placebo showed effect sizes of 0.31 and 0.37 (ref. ^16^). Similarly, comparison of change in CAPS score between paroxetine and placebo showed effect sizes of 0.56, 0.45 and 0.09 (ref. ^16^). By contrast, the effect size of 0.91 demonstrated in this study between MDMA-assisted therapy and placebo with therapy was larger than that for any other previously identified PTSD pharmacotherapy^16–18^. To directly assess superiority, a head-to-head comparison of MDMA-assisted therapy with SSRIs for PTSD would be needed. Although the present study tested the effects of MDMA using a model in which both treatment groups received supportive therapy, participants who received MDMA and supportive therapy (*d* = 2.1) had greater improvement in PTSD change scores compared with those who received placebo with supportive therapy (*d* = 1.2), suggesting that MDMA enhanced the effects of supportive therapy. In clinical practice, both MDMA and supportive therapy will be components of this PTSD treatment.

Previous research on MDMA for PTSD has suggested that those with a recent history of SSRI treatment may not respond as robustly to MDMA^18^. Given that 65.5% of participants in the current trial have a lifetime history of SSRI use, it is difficult to separate the ramifications of long-term SSRI treatment from the effects of treatment resistance. However, there was no obvious effect of previous SSRI use on therapeutic efficacy in this trial. Similarly, although years of PTSD diagnosis or age of onset may affect treatment efficacy, no obvious relationship was seen here between duration or onset of PTSD diagnosis and treatment efficacy.

Serotonin and the serotonin transporter are of particular importance in the generation, consolidation, retrieval and reconsolidation of fear memories^19,20^. Reduced serotonin transporter levels (which result in greater amounts of extracellular serotonin) have been shown to predict propensity to develop PTSD^21^, increase fear and anxiety-related behaviors^22^, and induce greater amygdalar blood oxygenation level-dependent (BOLD) activity in response to fearful images^23^. There is extensive serotonergic innervation of the amygdala, and amygdalar serotonin levels have been shown to increase following exposure to stressful and fear-inducing stimuli^24^. MDMA enhances the extinction of fear memories in mice through increased expression of brain-derived neurotrophic factor in the amygdala, and human neuroimaging studies have demonstrated that MDMA is associated with attenuated amygdalar BOLD activity during presentation of negative emotional stimuli^25^. Together these data suggest that MDMA may exert its therapeutic effects through a well-conserved mechanism of amygdalar serotonergic function that regulates fear-based behaviors and contributes to the maintenance of PTSD. Perhaps by reopening an oxytocin-dependent critical period of neuroplasticity that typically closes after adolescence^15^, MDMA may facilitate the processing and release of particularly intractable, potentially developmental, fear-related memories.

It is intriguing to speculate that the pharmacological properties of MDMA, when combined with therapy, may produce a ‘window of tolerance,’ in which participants are able to revisit and process traumatic content without becoming overwhelmed or encumbered by hyperarousal and dissociative symptoms^26^. MDMA-assisted therapy may facilitate recall of negative or threatening memories with greater self-compassion^27^ and less PTSD-related shame and anger^28^. Additionally, the acute prosocial and interpersonal effects of MDMA^25,29^ may support the quality of the therapeutic alliance, a potentially important factor relating to PTSD treatment adherence^30^ and outcome^31^. Indeed, clinicians have suggested that “MDMA may catalyze therapeutic processing by allowing patients to stay emotionally engaged while revisiting traumatic experiences without becoming overwhelmed“^32^.

Given that PTSD is a strong predictor of disability in both veteran and community populations^33^, it is promising to note that the robust reduction in PTSD and depressive symptoms identified here is complemented by a significant improvement in SDS score (for example, work and/or school, social and family functioning). Approximately 4.7 million US veterans report a service-related disability^34^, costing the US government approximately $73 billion per year^35^. Identification of a PTSD treatment that could improve social and family functioning and ameliorate impairment across a broad range of environmental contexts could provide major medical cost savings, in addition to improving the quality of life for veterans and others affected by this disorder.

PTSD is a particularly persistent and incapacitating condition when expressed in conjunction with other disorders of mood and affect. In the present study, perhaps most compelling are the data indicating efficacy in participants with chronic and severe PTSD, and the associated comorbidities including childhood trauma, depression, suicidality, history of alcohol and substance use disorders, and dissociation, because these groups are all typically considered treatment resistant^2–6^. Given that more than 80% of those assigned a PTSD diagnosis have at least one comorbid disorder^3^, the identification of a therapy that is effective in those with complicated PTSD and dual diagnoses could greatly improve PTSD treatment. Additional studies should therefore be conducted to evaluate the safety and efficacy of MDMA-assisted therapy for PTSD in those with specific comorbidities.

Although recent research suggests that dissociative subtype PTSD is difficult to treat^36^, participants with the dissociative subtype who received MDMA-assisted therapy had significant symptom reduction that was at least similar to that of their counterparts with non-dissociative PTSD. Given that this covariate was significant, it warrants further study. Furthermore, given that other treatments for PTSD are not consistently effective for those with the dissociative subtype, these data, if replicated, would indicate an important novel therapeutic niche for MDMA-assisted therapy for typically hard-to-treat populations.

Importantly, there were no major safety issues reported in the MDMA arm of this study. Although abuse potential, cardiovascular risk and suicidality were recorded as AESIs, MDMA was not shown to induce or potentiate any of these conditions. In addition, although there was often a transient increase in blood pressure during MDMA sessions, this was expected based on phase 2 data and previous studies in healthy volunteers^37^. These data suggest that MDMA has an equivalent, if not better, safety profile compared with that of first-line SSRIs for the treatment of PTSD, which are known to carry a low risk of QT interval prolongation^38^.

There are several limitations to the current trial. First, due to the coronavirus disease 2019 (COVID-19) pandemic, the participant population is smaller than originally planned. However, given the power noted in this study, it is unlikely that population size was an impediment. Second, the population is relatively homogeneous and lacks racial and ethnic diversity, which should be addressed in future trials. Third, this report describes the findings of a short-term pre-specified primary outcome, 2 months after the last experimental session and 5 weeks since the final integrative therapy session; long-term follow-up data from this controlled trial will be collected to assess durability of treatment. Fourth, safety data were by necessity collected by site therapists, perhaps limiting the blinding of the data. To eliminate this effect on the primary and secondary outcome measures, all efficacy data were collected by blinded, independent raters. Last, given the subjective effects of MDMA, the blinding of participants was also challenging and possibly led to expectation effects^14^. However, although blinding was not formally assessed during the study, when participants were contacted to be informed of their treatment assignment at the time of study unblinding it became apparent that at least 10% had inaccurately guessed their treatment arm. Although anecdotal, at least 7 of 44 participants in the placebo group (15.9%) inaccurately believed that they had received MDMA, and at least 2 of 46 participants in the MDMA group (4.3%) inaccurately believed that they had received placebo.

We may soon be confronted with the potentially enormous economic and social repercussions of PTSD, exacerbated by the COVID-19 pandemic. Overwhelmingly high rates of psychological and mental health impairment could be with us for years to come and are likely to impart a considerable emotional and economic burden. Novel PTSD therapeutics are desperately needed, especially for those for whom comorbidities confer treatment resistance.

In summary, MDMA-assisted therapy induces rapid onset of treatment efficacy, even in those with severe PTSD, and in those with associated comorbidities including dissociative PTSD, depression, history of alcohol and substance use disorders, and childhood trauma. Not only is MDMA-assisted therapy efficacious in individuals with severe PTSD, but it may also provide improved patient safety. Compared with current first-line pharmacological and behavioral therapies, MDMA-assisted therapy has the potential to dramatically transform treatment for PTSD and should be expeditiously evaluated for clinical use.

## Methods

### Study design

This was a randomized double-blind study designed to compare the efficacy of MDMA-assisted therapy with that of placebo with therapy. Fifteen study sites, consisting of 11 in the United States, two in Canada and two in Israel, included both institutional sites and private clinics. Ethics approval was obtained from Copernicus Group Independent Review Board, Western Institutional Review Board, University of British Columbia Providence Healthcare Research Ethics Board, and the Helsinki Committees of Be’er Ya’akov Ness Ziona Mental Health Center and Chaim Sheba Medical Center. This clinical study was conducted in accordance with the principles of the Declaration of Helsinki. The public study protocol is available at http://maps.org/mapp1. The therapist manual is available at http://maps.org/treatment-manual.

### Participants

Participants were recruited through print and internet advertisements, referrals from treatment providers, and by word of mouth. Participants were required to initiate contact with the study sites themselves, even if recommended by a provider. After providing written informed consent, participants were screened for eligibility. The criteria for inclusion consisted of meeting the *Diagnostic and Statistical Manual of Mental Disorders*, fifth edition (DSM-5) criteria for current PTSD with a symptom duration of ≥6 months at screening (as assessed with the Mini International Neuropsychiatric Interview (MINI) for DSM-5), and a CAPS-5 total severity score of ≥35 at baseline. Exclusion criteria consisted of primary psychotic disorder, bipolar I disorder, dissociative identity disorder, eating disorders with active purging, major depressive disorder with psychotic features, personality disorders, current alcohol and substance use disorders, pregnancy or lactation, and any medical condition that could make receiving a sympathomimetic drug harmful due to increased blood pressure and heart rate, including uncontrolled hypertension, history of arrhythmia, or marked baseline prolongation of QT and/or QTc interval. Participants with other mild, stable, chronic medical problems (for example, type 2 diabetes mellitus or well-controlled hypertension) were eligible for enrollment if the site physician, clinical investigator and medical monitor agreed that the condition would not increase the risk associated with MDMA administration. Participants were required to comply with lifestyle modifications, including a medically supervised discontinuation of psychiatric medications for a minimum of five half-lives plus one additional week before the baseline assessments (see the study protocol for inclusion and exclusion criteria).

The study protocol was amended on three occasions during study enrollment: first, to add clarity to eligibility criteria related to comorbid medical conditions; second, to add terms of suicidal ideation and behavior as AESIs, as requested by the FDA; and third, to increase the frequency of suicidality assessments following experimental sessions, as requested by the FDA, and to add an option for some telemedicine visits following the COVID-19 pandemic. Given that the study was at full enrollment (*n* = 105) when COVID-19 shut down in-person interactions at most of the study sites, the FDA and sponsor concluded that a reduced sample size of 90 participants, instead of the planned 100, would maintain sufficient statistical power to meet study objectives and would avoid COVID-19 delays of experimental sessions, which might confound the assessment of treatment effects.

### Study drug

The study drug was manufactured in accordance with Current Good Manufacturing Practice (CGMP) standards by Onyx Scientific and compounded by Sharp Clinical Services. Assays for chemistry, manufacturing and controls were completed in accordance with the CGMP and International Council for Harmonisation of Technical Requirements for Pharmaceuticals for Human Use (ICH) standards, and reported to the FDA, Health Canada and the Israel Ministry of Health.

### Randomization, masking and bias minimization

Participants were randomized in a blinded fashion and were allocated 1:1 to either the MDMA-assisted therapy group or the placebo with therapy group. Randomization was stratified by site and occurred following enrollment confirmation (after preparatory visits). Randomization was managed via an interactive web randomization system—ITClinical IWRS, version 11.0.1 (ITClinical, LDA)—based on a centralized randomization schedule developed by an independent third-party vendor to maintain blinding. Participants, site staff and the sponsor were blinded to participant group assignment until after the database was locked.

An inactive placebo with therapy was utilized as the comparator to isolate the efficacy of the MDMA itself. Although low-dose MDMA improved blinding in phase 2 studies, it led to decreased effectiveness compared with an inactive placebo in a PTSD population, making it easier to detect a difference between the active and comparator groups^15^. The use of inactive placebo also allows for uncontaminated comparison of safety data between groups. Therefore, an inactive placebo was determined in partnership with the FDA as a more conservative statistical comparison, and the study utilized observer-blinded efficacy assessments to minimize bias in efficacy measurements.

An observer-blind and centralized independent rater pool was used to administer the primary and secondary outcome measures, that is, the CAPS-5 and the SDS for functional impairment, the latter of which was adapted to limit missing item-level data as per the FDA requirements and included use of the three-item mean as the total score and imputation of work-related impairment as the maximum score, if caused by PTSD. The independent rater measurements were conducted at baseline and following each experimental session via live video interviews. Independent raters did not repeatedly see the same participant and the independent rater pool was blinded to the complete study design, visit number, treatment assignment, and all data collected by the therapy team after baseline, with the exception of safety data related to suicidality. Participants were instructed to withhold their opinion on treatment group assignment from independent raters and to refrain from sharing details regarding the study design and their number of completed visits. To ensure that all site and sponsor staff were shielded from study outcome measures, primary and secondary outcome measures were collected from the blinded independent rater pool and stored in a dedicated database that was separate from the blinded, clinical database.

### Procedures

Following an initial phone screening, participants provided written informed consent and underwent further screening assessments for eligibility. These included the PTSD Checklist for DSM-5 (PCL-5), the MINI for DSM-5, the Structured Clinical Interview for DSM-5 Screening Personality Questionnaire and Structured Clinical Interview for DSM-5 Personality Disorders (SCID-5-SPQ and -PD), the Lifetime C-SSRS, medical history, and pre-study medications. Study staff contacted outside providers, ordered medical records, and conducted a physical examination, laboratory testing (including pregnancy and drug tests), electrocardiogram, and 1-min rhythm strip. Eligible participants were enrolled in the study and began psychiatric medication taper (Table 1) if needed, and collection of adverse events. Anticipated effects of MDMA, such as euphoria, stimulation and feelings of closeness^39^, were intentionally not solicited as adverse events to avoid biasing the collection of adverse event data. Participant medication taper was variable, lasting from 0 d (no taper needed) to 103 d. Clinical data were electronically captured using Medrio EDC versions R40–R40.7.

In accordance with FDA guidance, we paid special attention to a subset of adverse events, termed AESIs, relating to cardiac function that could be indicative of QT interval prolongation or cardiac arrhythmias, abuse liability, and suicidal ideation and behavior. All adverse events that included signs or symptoms potentially associated with a cardiovascular event such as palpitations or dizziness were further evaluated for reporting as a cardiovascular adverse event. To assess signs of MDMA abuse potential, any adverse event terms such as ‘behavioral addiction’, ‘drug abuser’, ‘substance abuser’, ‘dependence’, ‘intentional product misuse’, ‘overdose’ (accidental, intentional or prescribed) or ‘drug diversion’ were collected and coded as AESIs. Suicidal ideation that was judged as serious or severe by the investigator, serious ideation defined as a C-SSRS suicidal ideation score of a 4 or 5, self-harm in the context of any suicidal ideation, and any suicide attempts were reported as AESIs.

Enrolled participants underwent three 90-min preparatory sessions of therapy with a two-person therapist team in preparation for experimental sessions (Fig. 1). The preparatory sessions focused on establishing therapeutic alliance and trust, and also provided guidance on how to respond to the memories and feelings that could arise during treatment. Participants who failed to meet all eligibility criteria were withdrawn during this preparatory period. Baseline CAPS-5 assessment (to confirm PTSD diagnosis and total severity score of ≥ 35 for randomization) was performed by the independent rater pool after completion of two preparatory sessions and any necessary psychiatric medication taper to establish baseline symptom severity following removal of psychiatric medications. At the end of the preparatory period, participants were assessed for final eligibility and enrollment was confirmed prior to randomization (Fig. 1).

The treatment period consisted of three 8-h experimental sessions of either MDMA-assisted therapy or therapy with inactive placebo control, spaced ~4 weeks apart. Following a 10-h fast, experimental sessions began with a qualitative urine drug screen, pregnancy screen if applicable, and C-SSRS, as well as measurement of baseline blood pressure, body temperature and heart rate immediately before the initial drug dose. Any positive findings on the urine drug screen that could not be attributed to pre-approved concomitant medications were reviewed by the medical monitor to assess compliance with ongoing eligibility criteria and for possible AESIs. Experimental sessions were conducted following a circadian rhythm-adjusted dosing schedule for a morning (~10:00 hours) initial dose.

In each experimental session the participants received a single divided dose of 80–180 mg MDMA or placebo. In the first experimental session, an initial dose of 80 mg was followed by a supplemental half-dose of 40 mg 1.5–2.5 h after the first dose. In the second and third experimental sessions, an initial dose of 120 mg was followed by a supplemental half-dose of 60 mg. If tolerability issues emerged with the initial dose or if participants declined, the protocol permitted the supplemental dose and/or dose escalation to be withheld. There were no instances in which the supplemental dose was withheld due to tolerability issues. Six participants chose either not to take the supplemental dose (*n* = 3, 1 MDMA) or not to escalate to the 120 mg dose (*n* = 3, 2 MDMA) in a total of six experimental sessions (2.3% of the total sessions across the study). Blood pressure, body temperature and heart rate were measured before the supplemental dose was given^14^.

Manualized therapy was conducted in accordance with the MDMA-assisted therapy treatment manual (http://maps.org/treatment-manual). Therapy was inner-directed and designed to invite inquiry and to facilitate therapeutic effect by providing support for approaching difficult material in a manner that would not interfere with the participant’s spontaneous experience. Every therapist held a Master’s degree or above, and the protocol requirement was that one person per therapy team was licensed to provide psychotherapy in accordance with state and local requirements. Therapists were additionally required to take part in the sponsor’s five-part training process, which consisted of an online course (15 h), a training course (5 d), experiential learning (3 d), role playing (1 d), and supervision (52 h).

Blood pressure, body temperature and heart rate were measured at the end of each experimental session prior to discharging the participant.

Each experimental session was followed by three 90-min integration sessions that were spaced ~1 week apart to allow the participant to understand and incorporate their experience. The first integration session always occurred on the morning after the experimental session, and the remaining two integration sessions occurred over the following 3–4 weeks (Fig. 1).

Independent raters conducted CAPS-5 and SDS assessments ~3 weeks after each of the first two experimental sessions. The primary outcome assessment was conducted ~8 weeks after the third experimental session (18 weeks after the baseline assessment), in which the independent raters collected the final CAPS-5 and SDS assessments. Twenty per cent of independent rater assessments were randomly selected and reviewed for fidelity. Lead independent raters evaluated the fidelity of all assessments related to enrollment failures as well as an additional 20% of remaining baseline CAPS-5 assessments. Diagnostic concordance between the raters had a Cohen’s kappa coefficient of 0.94, and reliability analysis of the CAPS-5 total severity scores showed a Spearman correlation coefficient of 0.98 (*P* < 0.0001), demonstrating high inter-rater reliability between the independent raters. The independent raters were all mental health professionals with graduate-level training in psychology, social work or counseling, at least 1 year of experience working with trauma-exposed populations, and had previous experience administering structured assessments.

Cases of non-compliance, protocol deviations, loss to follow-up, and other reasons for participant dropout were assessed for the presence of AESIs. There were two major protocol deviations (defined as the eligibility criteria not being met by the randomized participants during the course of the study). In the first protocol deviation a participant was not compliant with drug use lifestyle modifications on study, and in the second protocol deviation a participant disclosed cannabis use at study entry but abstained for the duration of the study. There was one dosing error in which a participant in the placebo group received 80 mg placebo as an initial dose and 100 mg as a supplemental dose (*n* = 1). Additionally, 14 participants (10 of whom were in the MDMA arm) requested further integrative visits, as permitted by the protocol.

### Objectives

The primary objective of this trial was to evaluate the efficacy and safety of MDMA-assisted therapy for PTSD compared with placebo with therapy, based on comparison of CAPS-5 total severity score at baseline with that at 18 weeks after baseline. The CAPS-5 is a semi-structured interview that assesses the index history of DSM-5-defined traumatic event exposure, including the most distressing event, to produce a diagnostic score (presence versus absence) and a PTSD total severity score. The CAPS-5 rates intrusion symptoms (intrusive thoughts or memories), avoidance, cognitive and mood symptoms, arousal and reactivity symptoms, duration and degree of distress, and dissociation. The CAPS-5 is scored on a scale from 0 to 80, with moderate PTSD defined from a rationally derived severity range of 23–34 (ref. ^40^), and severe PTSD as ≥35.

The secondary objective of this trial was to evaluate the efficacy of MDMA-assisted therapy for PTSD compared with placebo with therapy in clinician-rated functional impairment, as measured by the mean change in SDS total scores from baseline to 18 weeks after baseline. Exploratory outcome measures included the BDI-II, the Alcohol Use Disorders Identification Test (AUDIT), the Drug Use Disorders Identification Test (DUDIT) and the Adverse Childhood Experiences (ACE) Questionnaire.

### Follow-up

Participants agreed to be recontacted for potential enrollment in a long-term follow-up study, which will include follow-up measures to assess the durability of the treatment. These data will be published at a later date.

### Statistics and reproducibility

Statistical power calculations for the initial sample size were made by fitting an MMRM of CAPS-4 data (converted to the CAPS-5 scale and pooled from the phase 2 studies) to obtain variance and covariance parameter estimates. Using the estimated effect size and variance and covariance parameters, the sample size was calculated to achieve a power of 90% at an alpha of 0.049.

The intent-to-treat (ITT) set consisted of 91 randomized participants, however, one participant declined dosing on the morning of the session and provided no additional data, and therefore it was not possible to complete this analysis. Participants were randomized in a blinded fashion with 1:1 allocation as described in the section on randomization, masking and bias minimization above. The modified intent-to-treat (mITT) set consisted of 90 randomized participants who had completed at least one blinded experimental session and at least one post-treatment assessment. The mITT set consisted of 46 participants randomized to the MDMA group and 44 participants randomized to the placebo group, with identical therapy. The per protocol set (completers) consisted of all participants who completed three experimental sessions and assessments (MDMA, *n* = 42; placebo, *n* = 37) (Fig. 1).

The SAP was guided by the ICH E9 (R1) guidelines, which describe the use of estimands and sensitivity analyses to measure the effects of the drug if taken as directed (de jure, assessment of efficacy), and the effects of the drug if taken as assigned, regardless of adherence (de facto, assessment of effectiveness). The SAP was developed in accordance with FDA requirements and was approved by the European Medicines Agency to meet the requirements for future marketing applications. The primary and secondary efficacy analyses therefore utilized a de jure estimand of the mITT set for assessing treatment efficacy from the CAPS-5 and SDS data while on the study drug. The de jure dataset did not include outcome measurements taken after treatment discontinuation in the analysis of treatment efficacy. Missing data were not imputed.

One participant in the placebo group completed only the baseline assessment, and discontinued intervention but provided CAPS data at the T4 timepoint, ~18 weeks after baseline. Given that no endpoint assessment was collected prior to treatment discontinuation, this participant is excluded from the de jure estimand (leaving *n* = 89) but is included in the de facto estimand sensitivity analysis (for a total of *n* = 90). Two additional CAPS data points at the T4 timepoint, ~18 weeks after baseline, from two participants in the placebo group who provided these data following discontinuation of treatment, were not included in the de jure estimand (Supplementary Table 1).

The de facto estimand assessed the impact of these missing data points in the mITT set. That is, the CAPS measures at the T4 timepoint, ~18 weeks after baseline for the three placebo participants who discontinued treatment but provided off-treatment outcome assessments were included in a sensitivity analysis, which determined that inclusion of these measures in the model did not significantly alter the results.

The primary and secondary efficacy analyses were carried out using an MMRM that included all outcome data from baseline and the first, second and third experimental sessions. The efficacy of treatment was tested by comparing the change from baseline to the third experimental session in CAPS-5 and SDS scores between treatment groups in two-sided tests. The fixed effects were treatment (MDMA or placebo), baseline CAPS score, dissociative subtype and investigational site, with random effect specified as study participant.

A hierarchical testing strategy was used to control for type I error, such that the hypothesis for the key secondary endpoint (SDS) would be tested only if the statistical test for the primary efficacy comparison rejected the null hypothesis. An analysis of covariance (ANCOVA) to test the effects of study participation before versus after the COVID-19 pandemic declaration by the World Health Organization indicated a non-significant interaction and therefore was not included in the primary outcome model (Supplementary Table 2). The primary outcome analysis was replicated independently by one blinded programmer and one unblinded programmer.

An independent data monitoring committee monitored adverse events for safety and conducted one administrative interim analysis, after completion of enrollment and of 60% of primary endpoints to examine the adequacy of the sample size. The data monitoring committee recommended that no additional participants should be added, based on conditional power calculations supporting 90% statistical power, but in keeping with the SAP did not provide the sponsor with any information on the conditional power or effect size. The alpha level was set to 0.05, and 2% of the alpha (0.001) was spent on the interim analysis and 98% (0.0499) was left for the final analysis.

Statistics for the primary and secondary efficacy comparisons (CAPS and SDS) are reported as *P* values from the results of the MMRM analysis. In exploratory analyses, additional baseline covariates of age, gender, ethnicity, prior use of SSRIs, depression as measured by the BDI-II, adverse childhood experiences, and alcohol and substance use disorders were assessed in the model, with the threshold of significance set at *P* < 0.05 (Supplementary Table 1). BDI-II score was also assessed as an exploratory efficacy outcome measure with a paired, two-tailed *t*-test. Results are reported as mean (s.d.) throughout the text. Between-group effect size was calculated with Cohen’s *d*, and 95% CIs are reported. SAS version 9.4 (SAS Institute) was used for analyses.

The safety analysis included all participants who were given at least one dose of the study drug or placebo. The primary safety analysis evaluated TEAEs as a participant-level analysis. An association with MDMA was determined based on the relative incidence of TEAEs with at least a twofold difference between the MDMA and placebo groups.

### Reporting Summary

Further information on research design is available in the Nature Research Reporting Summary linked to this article.

## Online content

Any methods, additional references, Nature Research reporting summaries, source data, extended data, supplementary information, acknowledgements, peer review information; details of author contributions and competing interests; and statements of data and code availability are available at 10.1038/s41591-021-01336-3.

## Supplementary information

Supplementary Information Supplementary Tables 1–4.

Reporting Summary

## Data Availability

The data that support the findings of this study are available from the sponsor (MAPS). However, restrictions apply to the availability of these data, which were used under license for the current study, and so are not publicly available. Data are, however, available from the authors upon reasonable request and with the permission of MAPS at http://maps.org/datause. All requests for raw and analyzed data are promptly reviewed by the sponsor delegate and trial organizer, MAPS PBC, to verify if the request is subject to any confidentiality obligations. Patient-related data not included in the paper were generated as part of clinical trials and may be subject to patient confidentiality. Any data that can be shared will be released via a data use agreement.

## Code availability

Commercially available software (SAS version 9.4, SAS Institute) was used for analyses, in keeping with the SAP.
