# Supplementary material for: MDMA-assisted therapy for severe PTSD: a randomized, double-blind, placebo-controlled phase 3 study
Source: Nat Med. 2021 May 10;27(6):1025–33. doi: 10.1038/s41591-021-01336-3 (PMC8205851; doi:10.1038/s41591-021-01336-3)
Supplement: Supplementary file 1 — Supplementary Tables 1–4. [file 41591_2021_1336_MOESM1_ESM.pdf]

nature portfolio

https://doi.org/10.1038/s41591-021-01336-3

Supplementary information

MDMA-assisted therapy for severe PTSD: a randomized, double-blind, placebo-controlled phase 3 study

In the format provided by the authors and unedited

SUPPLEMENTARY DATA TABLES

Supplementary Data Table 1. Missing Data from mITT Set Based on Reasons for Post-Randomization Withdrawal

**Post-randomization Events**	**Available Data Post-Withdrawal**	**Treatment Assignment**	**Summary of Missing Data and Inclusion in *de jure* Estimand by Visit**			
			Reason	Participant #	Missed Visits	*De jure estimand*
Discontinue Study, Withdraw Consent	No data	MDMA	COVID	0807[A]<br>1211[A]	T3, T4	Included T1, T2
			Perceived early efficacy	0611[B]	T3, T4	Included T1, T2
			Did not want to complete CAPS due to triggering (*primary*); AE- depressed mood (*secondary*)	0213[B]	T3, T4	Included T1, T2
		Placebo	SAE- suicidal ideation	0104[B]	T3, T4	Included T1, T2
			COVID	0707[A]	T3, T4	Included T1, T2
			Choice	0711[B]	T3, T4	Included T1, T2
			AE- insomnia	0211	T4	Included T1, T2, T3
Discontinue Intervention (or experiences long delay) and does not receive Rescue Medication	Data collection continues whenever possible via remote visits	MDMA	None Observed			
		Placebo	COVID[A]	1502[A]	None	Included T1, T2, T3
Discontinue Intervention (or experiences long delay) and receives Rescue Medication	Data collection continues whenever possible via remote visits	MDMA	None Observed			
		Placebo	SAE- suicide attempt	0203	T3	Included T1, T2
			AE- increased anxiety	0102	T2, T3	*Excluded* [C]
Death	No data	MDMA	None Observed			
		Placebo	None Observed			
Suicide	No data	MDMA	None Observed			
		Placebo	None Observed			

[A] COVID, participants for which participation was terminated due to study closure related to the COVID-19 pandemic

[B] Discontinued participation and withdrew consent which led to missing data.

[C] Participant completed only Baseline T1, discontinue intervention and received rescue medication, and T4. As no endpoint assessment was collected prior to treatment discontinuation, this participant is excluded from the *de jure* estimand but included in the *de facto* estimand sensitivity analysis.

Supplementary Data Table 2. Analysis Effects on Primary Results

MMRM Results for Fixed Effects		
**Effect**	**F-Value**	**Pr > F**
Treatment	12.6	0.0007
Baseline CAPS-5 Score	0.13	0.7219
Study Visit	51.54	<.0001
Treatment x Study Visit	8.75	0.0004
Dissociative Subtype	2.03	0.159
Study Site	1.6	0.1003
**Analysis of Covariate Effects on Primary Results**		
**Variable**	**p-value main effect**	**p-value interaction w/Tx**
Age (continuous)	0.2491	0.8291
Sex biological	0.6281	0.9376
Disabled (yes/no)	0.9883	0.4444
Covid-19 (pre/during)	0.7701	0.9633
Prior SSRI Usage (yes/no)	0.6114	0.7650
PTSD Duration (continuous)	0.6688	0.3795
Dissociative Subtype (yes/no)	0.0350	0.0044
BDI (>=23)	0.1003	0.6373
ACE (>=4)	0.5198	0.5127
AUDIT (>=5)	0.4975	0.4071
DUDIT (>=5)	0.6420	0.6441

Supplementary Data Table 2: Each of these covariates were added one-at-a-time as fixed effects to the primary efficacy model and analyzed as 2-sided F-tests without correction for multiple comparisons. The p-values associated with their coefficient estimates are reported.

Supplementary Data Table 3: Treatment Emergent Adverse Events Related to MDMA

Adverse Event (PT)	MDMA-assisted therapy (n=46)	Placebo with therapy (n=44)
Muscle Tightness	29 (63.0%)	5 (11.4%)
Decreased Appetite	24 (52.2%)	5 (11.4%)
Nausea	14 (30.4%)	5 (11.4%)
Hyperhidrosis	9 (19.6%)	1 (2.3%)
Feeling Cold	9 (19.6%)	3 (6.8%)
Restlessness	7 (15.2%)	0
Mydriasis	7 (15.2%)	0
Dizziness Postural	6 (13.0%)	2 (4.5%)
Bruxism	6 (13.0%)	1 (2.3%)
Nystagmus	6 (13.0%)	0
Blood Pressure Increased	5 (10.9%)	0
Feeling Jittery	5 (10.9%)	0
Non-Cardiac Chest Pain	5 (10.9%)	1 (2.3%)
Dry Mouth	5 (10.9%)	2 (4.5%)
Vision Blurred	4 (8.7%)	1 (2.3%)
Pollakiuria	4 (8.7%)	1 (2.3%)
Intrusive Thoughts	4 (8.7%)	0
Vomiting	4 (8.7%)	0
Stress	4 (8.7%)	0
Musculoskeletal Pain	4 (8.7%)	0
Pyrexia	3 (6.5%)	1 (2.3%)
Chills	3 (6.5%)	0
Substance Use (*cannabis)*	3 (6.5%)	0
Micturition urgency	3 (6.5%)	0
Muscle Twitching	3 (6.5%)	0
Somnolence	3 (6.5%)	0
Nervousness	3 (6.5%)	0

Supplementary Data Table 3. Treatment Emergent Adverse Events (TEAEs). AEs that occurred from the first experimental session to study termination based on participant level incidence were termed TEAE. Relationship to MDMA was determined based on relative incidence of TEAEs with at least two-fold difference between MDMA vs. placebo. Listed here are the most common (>5% of subjects) TEAEs related to MDMA.

Supplementary Data Table 4: Changes in Vital Signs During the Experimental Sessions

	MDMA (n = 46)	Placebo (n=44)	Total (n=90)
**Experimental Session 1**			
Predose (n)	46	44	90
Systolic, mean (SD)	126.4 (18.55)	120.2 (13.92)	123.4 (16.65)
Diastolic, mean (SD)	79.4 (11.23)	79.6 (8.76)	79.5 (10.04)
Pulse (beats per minute), mean (SD)	71.0 (11.16)	73.2 (11.20)	72.0 (11.17)
Body temperature (degrees C), mean (SD)	36.6 (0.49)	36.6 (0.39)	36.6 (0.44)
Interim (n)	46	44	90
Systolic, mean (SD)	139.1 (19.70)	122.3 (14.95)	130.9 (19.37)
Diastolic, mean (SD)	85.3 (10.41)	79.1 (11.38)	82.3 (11.28)
Pulse (beats per minute), mean (SD)	82.3 (14.49)	69.6 (11.57)	76.1 (14.56)
Body temperature (degrees C), mean (SD)	36.9 (0.42)	36.7 (0.36)	36. 8 (0.40)
Endpoint (n)	46	44	90
Systolic, mean (SD)	130.8 (16.63)	119.2 (14.77)	125.1 (16.71)
Diastolic, mean (SD)	81.7 (9.63)	77.2 (11.17)	79.5 (10.60)
Pulse (beats per minute), mean (SD)	79.4 (12.51)	73.7 (10.13)	76.6 (11.70)
Body temperature (degrees C), mean (SD)	36.8 (0.51)	36.9 (0.30)	36.8 (0.42)
**Experimental Session 2**			
Predose (n)	43	41	84
Systolic, mean (SD)	127.2 (14.46)	117.0 (15.33)	122.2 (15.67)
Diastolic, mean (SD)	81.1 (10.35)	76.8 (9.52)	79.0 (10.13)
Pulse (beats per minute), mean (SD)	71.7 (10.44)	67.1 (10.28)	69.5 (10.55)
Body temperature (degrees C), mean (SD)	36.58 (0.44)	36.5 (0.41)	36.6 (0.42)
Interim (n)	43	41	84
Systolic, mean (SD)	146.5 (15.09)	118.1 (18.14)	132.7 (21.85)
Diastolic, mean (SD)	89.1 (10.39)	76.4 (12.45)	82.9 (13.02)
Pulse (beats per minute), mean (SD)	87.3 (12.80)	66.1 (10.61)	77.0 (15.84)
Body temperature (degrees C), mean (SD)	36.9 (0.46)	36.7 (0.30)	36.8 (0.40)
Endpoint (n)	43	41	84
Systolic, mean (SD)	128.8 (17.66)	118.9 (15.12)	123.9 (17.11)
Diastolic, mean (SD)	81.5 (12.36)	75.2 (9.02)	78.4 (11.25)
Pulse (beats per minute), mean (SD)	85.3 (14.66)	69.8 (8.75)	77.7 (14.35)
Body temperature (degrees C), mean (SD)	36.9 (0.48)	36.7 (0.29)	36.8 (0.41)
**Experimental Session 3**			
Predose (n)	42	37	79
Systolic, mean (SD)	128.5 (15.30)	118.0 (15.79)	123.6 (16.31)
Diastolic, mean (SD)	82.4 (8.00)	76.1 (10.98)	79.4 (9.96)
Pulse (beats per minute), mean (SD)	72.7 (11.80)	69.4 (11.49)	71.2 (11.70)
Body temperature (degrees C), mean (SD)	36.6 (0.46)	36.5585 (0.50)	36.5850 (0.47)
Interim (n)	41	37	78
Systolic, mean (SD)	145.9 (19.23)	117.9 (15.74)	132.6 (22.51)
Diastolic, mean (SD)	86.8 (10.32)	76.2 (10.07)	81.7 (11.45)
Pulse (beats per minute), mean (SD)	91.6 (16.95)	66.4 (12.99)	79.8 (19.75)
Body temperature (degrees C), mean (SD)	37.0 (0.47)	36.7 (0.325)	36.8 (0.44)
Endpoint (n)	42	37	79
Systolic, mean (SD)	127.6 (16.62)	117.5 (13.74)	122.9 (16.06)
Diastolic, mean (SD)	78.4 (11.15)	74.0 (9.39)	76.3 (10.53)
Pulse (beats per minute), mean (SD)	82.1 (14.95)	71.6 (11.84)	77.2 (14.49)
Body temperature (degrees C), mean (SD)	36.9 (0.50)	36.8 (0.40)	36.8 (0.45)

Supplementary Data Table 4. Changes in vital signs during the experimental sessions. Transient increases in blood pressure were observed in the MDMA group, as expected based on Phase 2 data and studies in healthy volunteers.
