# Supplementary material for: MDMA-assisted therapy for severe PTSD: a randomized, double-blind, placebo-controlled phase 3 study
Source: Nat Med. 2021 May 10;27(6):1025–33. doi: 10.1038/s41591-021-01336-3 (PMC8205851; doi:10.1038/s41591-021-01336-3)
Supplement: Supplementary file 2 — Reporting Summary [file 41591_2021_1336_MOESM2_ESM.pdf]

nature research

Corresponding author(s): Jennifer Mitchell, PhD

Last updated by author(s): Mar 31, 2021

Reporting Summary

Nature Research wishes to improve the reproducibility of the work that we publish. This form provides structure for consistency and transparency in reporting. For further information on Nature Research policies, see our Editorial Policies and the Editorial Policy Checklist.

Statistics

For all statistical analyses, confirm that the following items are present in the figure legend, table legend, main text, or Methods section.

n/a	Confirmed	
☐	☒	The exact sample size (*n*) for each experimental group/condition, given as a discrete number and unit of measurement
☐	☒	A statement on whether measurements were taken from distinct samples or whether the same sample was measured repeatedly
☐	☒	The statistical test(s) used AND whether they are one- or two-sided<br>*Only common tests should be described solely by name; describe more complex techniques in the Methods section.*
☐	☒	A description of all covariates tested
☐	☒	A description of any assumptions or corrections, such as tests of normality and adjustment for multiple comparisons
☐	☒	A full description of the statistical parameters including central tendency (e.g. means) or other basic estimates (e.g. regression coefficient) AND variation (e.g. standard deviation) or associated estimates of uncertainty (e.g. confidence intervals)
☐	☒	For null hypothesis testing, the test statistic (e.g. *F*, *t*, *r*) with confidence intervals, effect sizes, degrees of freedom and *P* value noted<br>*Give P values as exact values whenever suitable.*
☒	☐	For Bayesian analysis, information on the choice of priors and Markov chain Monte Carlo settings
☒	☐	For hierarchical and complex designs, identification of the appropriate level for tests and full reporting of outcomes
☐	☒	Estimates of effect sizes (e.g. Cohen's *d*, Pearson's *r*), indicating how they were calculated

Our web collection on statistics for biologists contains articles on many of the points above.

Software and code

Policy information about availability of computer code

Data collection	Commercially available software was used for data collection through the study including: electronic data capture (EDC) software (Medrio Version 40.5), and interactive web randomization system (IWRS) software (IT Clinical Version 11.0.1).
Data analysis	Commercially available software (SAS software version 9.4 (SAS Institute Inc., Cary, N.C.)) was used for analyses in keeping with the Statistical Analysis Plan.

For manuscripts utilizing custom algorithms or software that are central to the research but not yet described in published literature, software must be made available to editors and reviewers. We strongly encourage code deposition in a community repository (e.g. GitHub). See the Nature Research guidelines for submitting code & software for further information.

Data

Policy information about availability of data

All manuscripts must include a data availability statement. This statement should provide the following information, where applicable:

- Accession codes, unique identifiers, or web links for publicly available datasets
- A list of figures that have associated raw data
- A description of any restrictions on data availability

The data that support the findings of this study are available from the sponsor (MAPS). However, restrictions apply to the availability of these data, which were used under license for the current study, and so are not publicly available. Data are however available from the authors upon reasonable request and with permission of MAPS at maps.org/datause. All requests for raw and analyzed data are promptly reviewed by the sponsor delegate & trial organizer, MAPS Public Benefit Corporation to verify if the request is subject to any confidentiality obligations. Patient-related data not included in the paper were generated as part of clinical trials and may be subject to patient confidentiality. Any data that can be shared will be released via a Data Use Agreement.

Field-specific reporting

Please select the one below that is the best fit for your research. If you are not sure, read the appropriate sections before making your selection.

☒ Life sciences ☐ Behavioural & social sciences ☐ Ecological, evolutionary & environmental sciences

For a reference copy of the document with all sections, see nature.com/documents/nr-reporting-summary-flat.pdf

Life sciences study design

All studies must disclose on these points even when the disclosure is negative.

Sample size

Statistical power calculations for the initial sample size were made by fitting a mixed-effect repeated measure model (MMRM) of CAPS-4 data, converted to the CAPS-5 scale, pooled from the Phase 2 studies to obtain variance/covariance parameter estimates. Using the estimated effect size and variance/covariance parameters, the sample size was calculated to achieve a power of 90% at an alpha of 0.049.

Data exclusions

The intent-to-treat (ITT) set included n = 91 randomized participants, however one participant declined dosing on the morning of the session and provided no additional data, and therefore it was not possible to complete this analysis. The modified intent-to-treat (mITT) set included n = 90 randomized participants, defined as those who completed at least one blinded experimental session and at least one post-treatment assessment. The mITT set included a total of n = 46 participants randomized to MDMA and n = 44 to Placebo with identical therapy. The per protocol set (completers) included all participants who completed three experimental sessions and assessments (n = 42 MDMA, n = 37 Placebo). One placebo participant completed only Baseline T1, discontinued intervention but provided T4 CAPS data. As no endpoint assessment was collected prior to treatment discontinuation, this participant is excluded from the de jure estimand (leaving n = 89) but included in the de facto estimand sensitivity analysis (for a total of n = 90). Two additional T4 CAPS data points from placebo participants who provided this data following discontinuation of treatment were not included in the de jure estimand (see Supplementary Table 1).

Replication

This Phase 3 RCT replicates previous findings in a series of previously published controlled Phase 2 trials (Mithoefer 2019).

Randomization

Participants were randomized in a blinded fashion and 1:1 allocation to either the MDMA-assisted therapy group or the placebo with therapy group. Randomization was stratified by site and occurred following enrollment confirmation (after preparatory visits). Randomization was managed via an Interactive Web Randomization System (IWRS) based on a centralized randomization schedule developed by an independent third-party vendor to maintain blinding.

Blinding

Participants, site staff, and the sponsor were blinded to participant group assignment until after the database was locked. An observer-blind and centralized Independent Rater (IR) pool was used to administer the Primary and Secondary Outcome measures.

Reporting for specific materials, systems and methods

We require information from authors about some types of materials, experimental systems and methods used in many studies. Here, indicate whether each material, system or method listed is relevant to your study. If you are not sure if a list item applies to your research, read the appropriate section before selecting a response.

Materials & experimental systems

n/a	Involved in the study	
☒	☐	Antibodies
☒	☐	Eukaryotic cell lines
☒	☐	Palaeontology and archaeology
☒	☐	Animals and other organisms
☐	☒	Human research participants
☐	☒	Clinical data
☒	☐	Dual use research of concern

Methods

n/a	Involved in the study	
☒	☐	ChIP-seq
☒	☐	Flow cytometry
☒	☐	MRI-based neuroimaging

Human research participants

Policy information about studies involving human research participants

Population characteristics

Study arms were not significantly different in terms of race, ethnicity, sex, age, dissociative subtype, disability, and CAPS-5 score (see Table 1). The mean The mean duration of PTSD diagnosis was 14.8 (11.6) years and 13.2 (11.4) years in the MDMA and placebo groups, respectively. Of note, six participants in the MDMA group and 13 participants in the placebo group qualified as dissociative subtype per the CAPS-5.

Recruitment

Participants were recruited through print and internet advertisements, referrals from treatment providers, and by word of mouth. Participants were required to initiate contact with the study sites themselves, even if recommended by a provider. Since study participants often self-referred, self-selection bias must be considered. Participants may have been intrigued by the novelty or character of the therapeutic, may have had previous positive recreational experience with the therapeutic, or - since participants all were shouldering severe and sometimes dissociative PTSD - may have been willing to consider a

therapeutic that they may not have been willing to consider under less intractable circumstances. All participants provided written informed consent.

Ethics oversight	Ethics approval was obtained from Copernicus Group Independent Review Board, Western Institutional Review Board, University of British Columbia Providence Healthcare Research Ethics Board, and the Helsinki Committees of Beer Yaakov Ness Ziona Mental Health Center and Chaim Sheba Medical Center.

Note that full information on the approval of the study protocol must also be provided in the manuscript.

Clinical data

Policy information about clinical studies

All manuscripts should comply with the ICMJE guidelines for publication of clinical research and a completed CONSORT checklist must be included with all submissions.

Clinical trial registration	ClinicalTrials.gov NCT03537014
Study protocol	The public study protocol is available at maps.org/mapp1. To protect data integrity and study blind and to minimize bias, specific eligibility criteria and timing of assessments have been redacted from the public protocol.
Data collection	Fifteen study sites across the US (11), Canada (2), and Israel (2) included both institutional sites and private clinics. Participants were recruited from November 07, 2018 through May 26, 2020, with the last participant visit conducted on August 21, 2020. The final database was locked on October 27, 2020.
Outcomes	The Primary Outcome measure, the change in Clinician Administered PTSD Scale (CAPS-5), and the Secondary Outcome measure, the change in the Sheehan Disability Scale (SDS) were assessed by a blinded centralized Independent Rater (IR) pool multiple times throughout the study.
